# Development of an RTK-GPS Positioning Application with an Improved Position Error Model for Smartphones

**DOI:** 10.3390/s121012988

**Published:** 2012-09-25

**Authors:** Jinsang Hwang, Hongsik Yun, Yongcheol Suh, Jeongho Cho, Dongha Lee

**Affiliations:** 1 Department of Civil & Environmental Engineering, Sungkyunkwan University, Suwon 440-746, Korea; E-Mails: gpsboy@skku.edu (J.H.); jaycho@skku.edu (J.C.); 2 Department of Spatial Information Engineering, Pukyong National University, Busan 608-737, Korea; E-Mail: suh@pknu.ac.kr

**Keywords:** smartphone application, network RTK-GPS, NRTIP, Kalman filter, position error model

## Abstract

This study developed a smartphone application that provides wireless communication, NRTIP client, and RTK processing features, and which can simplify the Network RTK-GPS system while reducing the required cost. A determination method for an error model in Network RTK measurements was proposed, considering both random and autocorrelation errors, to accurately calculate the coordinates measured by the application using state estimation filters. The performance evaluation of the developed application showed that it could perform high-precision real-time positioning, within several centimeters of error range at a frequency of 20 Hz. A Kalman Filter was applied to the coordinates measured from the application, to evaluate the appropriateness of the determination method for an error model, as proposed in this study. The results were more accurate, compared with those of the existing error model, which only considered the random error.

## 1. Introduction

In cooperation with Dortmund University in Germany, the Federal Agency for Cartography and Geodesy (BKG) introduced HTTP-based Differential GPS (DGPS) data streaming technology and developed Networked Transport of RTCM via Internet Protocol (NTRIP) as the format of DGPS data [1]. Of the many types of DGPS data transferrable in the NTRIP format, the Network Real-Time Kinematic (NRTK) GPS data provides the most accurate real-time positioning. The NRTK positioning is a technology that calculates the correction message suitable for an area where a GPS rover is positioned [2,3]. The calculated correction message is transferred to the rover, to enable highly accurate real-time positioning [4]. Multiple GPS reference stations are utilized, because using a single reference station reduces accuracy if the distance between the rover and the reference station exceeds 10 km [5,6]. The NRTK correction message may be calculated using the data from multiple GPS reference stations placed at intervals of 50–70 km across a wide area, and the results may then be transferred in NTRIP format. The user can then conduct highly accurate positioning, using only a GPS rover and an NTRIP broadcaster connection terminal.

Representative NRTK services include the EUREF-IP service of the EU [7], SAPOS in Germany, the IGS-IP service of the International GNSS Service (IGS), and the National Virtual Reference Station (VRS) [8] Service of Korea. A mobile device connecting the Internet and a GPS receiver with a RTK processing function is required to use the NTRIP broadcaster. Mobile devices (e.g., PDAs, tablet PCs, and exclusive controllers) have conventionally been combined with a phone, for wireless Internet connection, and an expensive GPS receiver, with the RTK processing function, to organize a positioning system for NRTK. However, the recent development and distribution of new smartphones that support installation of user-developed application, high-speed processing, and wireless communication [9], provides a new environment for NTRIP-type NRTK services. Thus, most NRTK service features were enabled in the smartphone, which provides many advantages. With this background, this study now addresses the development of the NRTK application for smartphones.

The composition of the NRTK system was simplified, and the cost required to organize the system was reduced, by including the NTRIP client feature for correction message reception, NRTK processing feature, and applied features for using the NRTK positioning results in a smartphone application. A suitable method for determining the error model for the NRTK positioning results is also presented, to estimate highly reliable positioning values with a suitable filter in the application program, utilizing the NRTK positioning results.

## 2. NRTK Application Design for the Smartphone

This study designed the NRTK application using the advantages of the smartphone, including fast data processing speed, diverse wired/wireless communication features, and an installation environment for the universal user of the development application. Most of the processing for NRTK positioning could thus be conducted in the smartphone. This structure was applied for the following reasons. First, the system composition cost can be reduced, as the smartphone provides the RTK processing function, which until now was performed by the GPS receiver. Second, a smartphone performs all the functions that have been provided by a computer device, such as a PDA and a cell phone, simplifying the composition of the NRTK positioning system.

Table 1 shows the functions required for the application. The NRTK service with a virtual reference station, so called the virtual control point, was used for the NRTK service environment in Korea [10]. This method allows the user to transfer his rough position data to the NTRIP broadcaster, while the broadcaster calculates the correction message suitable for the user's position to be transferred to the user. Table 1 shows the core parts of the functions: communication and NRTK processing. The application used the Bluetooth-enabled device of the smartphone to control the GPS receiver, and receive the GPS observation results. The wireless communication module was used to provide the NTRIP client conducting the current position transfer and correction message reception, via the NTRIP broadcaster in the mobile environment. As for the NRTK processing function, the correction messages from communication devices and GPS observation data were processed. The precise positioning results were calculated within several centimeters of error range at a maximum frequency of 20 Hz.

**Figure 1.**
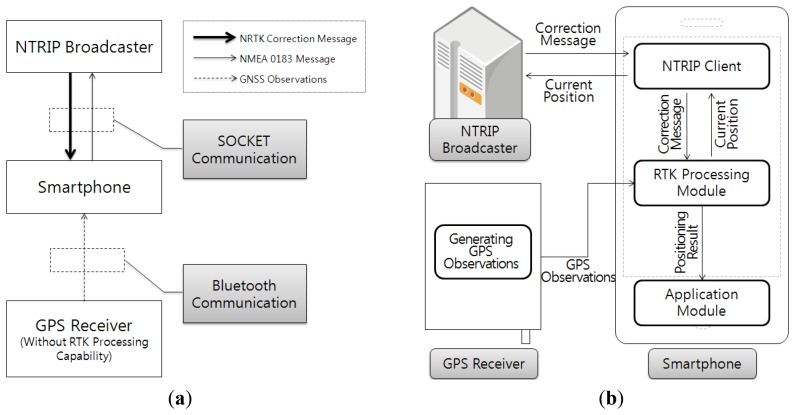
Design of the NRTK application for smartphones when use the general GPS receiver without the RTK function. (**a**) Communication method and data flow. **(b**) Composition of NRTK application and data flow.

Figure 1 shows the design results of the NRTK application for the smartphone, when the general GPS receiver without RTK processing function is used. Figure 1(a) shows the design of the communication method and data transfer among hardware devices that form the NRTK positioning system. Figure 1(b) shows the software design results, including the composition method of application software module, and data transfer among modules, GPS receiver, and NTRIP broadcaster.

Figure 1(b) shows that the application was organized in NTRIP client, NRTK processing, and utility module parts, so that the application could provide most functions needed for NRTK processing. This increases the processing load of the smartphone, but simplifies the role of the GPS receiver, to allow the GPS receiver without RTK processing function to form the NRTK positioning system.

**Figure 2.**
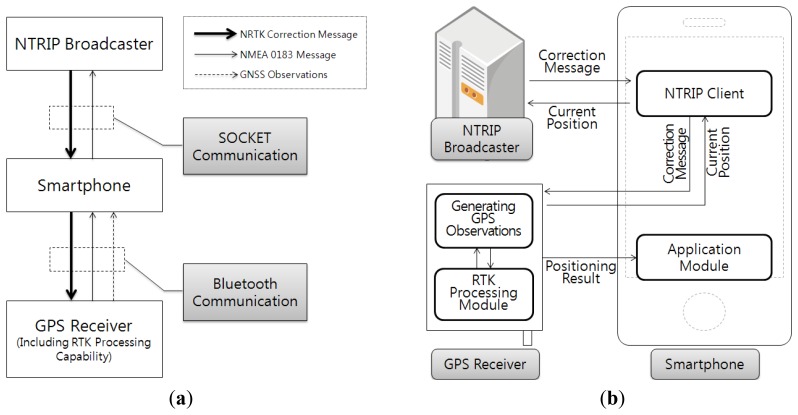
Design plan of the NRTK application for smartphones when use the expensive GPS receiver supporting the RTK function. (**a**) Communication method and data flow. (**b**) Composition of NRTK application and data flow.

Figure 2(a,b) shows the application design results, when an expensive GPS receiver with RTK processing function is used. Compared with the design results in Figure 1(a,b), most processing for NRTK positioning was concentrated on the GPS receiver in Figure 2. The NRTK application was composed so that it would only conduct the NTRIP client function, while it would apply module function using the coordinates calculated from the GPS receiver.

Considering the cost required for the system composition and equipment simplification, the design result in Figure 1 was suitable for the smartphone to conduct most of the processing. Based on these design results, an NRTK application for smartphones was developed in this study.

## 3. Application Development and Performance Evaluation

The NRTK application was developed in a smartphone equipped with the Android OS. Table 2 shows the development environment. The application was developed so that it operated with Android 2.2 (Froyo) or lower, using Android API (Level 8) and JDK 1.7 in Eclipse software, providing an integrated development environment. Java was used as the programming language for the development.

The application uses carrier-phase (L1 and L2) and pseudorange data as primary GPS observables and also uses correction messages enclosing correction information of all GPS observables for precise positioning. The supported formats of correction messages are RTCM v2.3, RTCM v3.0, and CMR.

**Figure 3.**
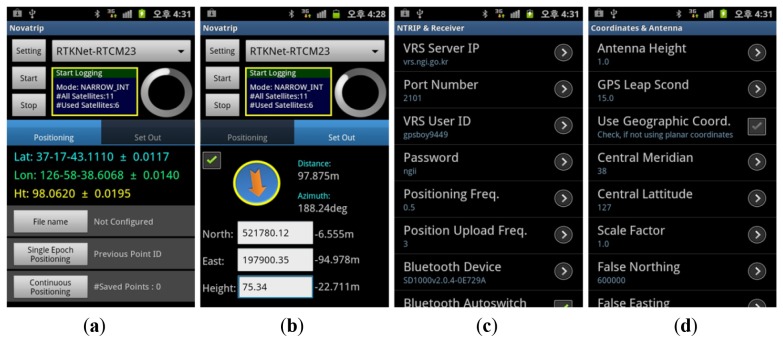
Application development results (main user interfaces and operation screens). (**a**) Application interface (Positioning). (**b**) Application interface (Set out). (**c**) Setting interface (NTRIP broadcast and GPS receiver). (**d**) Setting interface (Coordinate system and antenna).

Figure 3 shows the main user interfaces from the application development results. Figure 3(a,b) shows the application's utility functions serving as the user interfaces for positioning and field set out. Figure 3(c) shows the setting interface for the NTRIP broadcaster connection and GPS receiver control. Figure 3(d) shows the setting interface for the coordinate system and GPS antenna data. The development results showed that most of the functions for normal operation of the NRTK application could be conducted within the development environment of smartphones.

**Figure 4.**
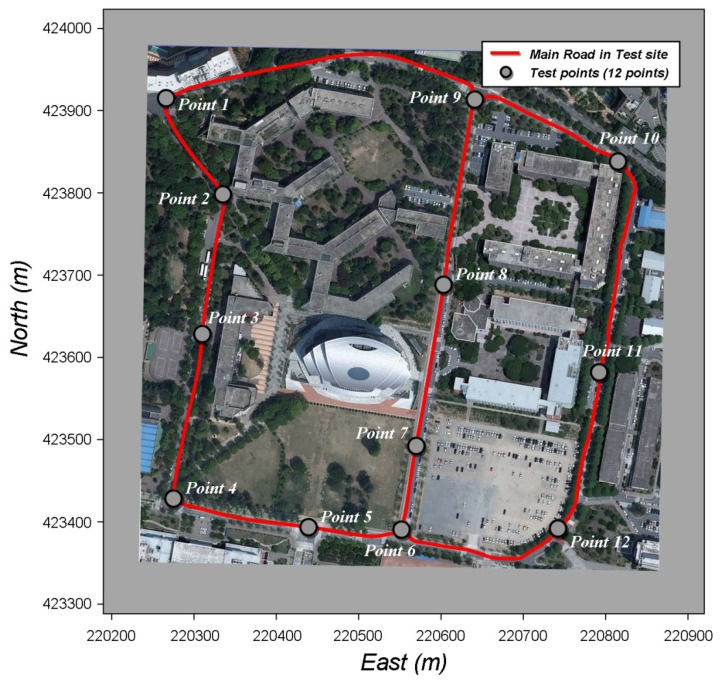
Test points for evaluating the performance of the NRTK application.

The most important of the functions of the NRTK application is the high accuracy and frequency positioning function. The normal accuracy levels for the RTK-GPS positioning method are 1 cm ± 1 ppm horizontally, and 2 cm ± 2 ppm vertically [11], with the maximum positioning frequency of 20 Hz [12]. This study developed the NRTK application with a performance level equivalent to such values. To evaluate the performance of the NRTK application, in terms of the positioning accuracy and frequency, the positions of twelve fixed points with accurately measured coordinates were measured for a long period. The statistical positioning accuracy and maximum frequency of the NRTK application were evaluated, by analyzing the test results based on measurements of these twelve points. The transfer time of correction message, that is, the latency of the correction message was analyzed, as it is among the main factors affecting the RTK-GPS positioning accuracy. Figure 4 shows the geographical distributions of test points for evaluating the performance of NRTK application. All points were selected along the main road with good environments of GPS signal reception.

**Figure 5.**
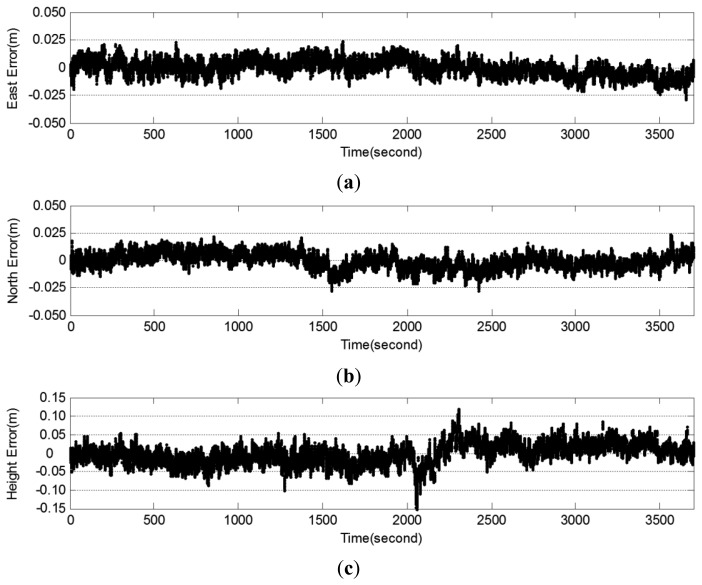
A sample of test results (at *Point 1*) for evaluating the positioning. (**a**) Positioning errors of north coordinates. (**b**) Positioning errors of east coordinates. (**c**) Positioning errors of ellipsoid heights.

Figure 5 shows the positioning accuracy of the NRTK application using the one-hour data at one fixed point as a sample. Figure 5(a–c) shows the time series data for the positioning errors of the north, east, and ellipsoid height coordinates, respectively. In most sections, the positioning error of horizontal coordinate (Figure 5(a,b)) was within a range of ±2.0 cm, and the positioning error of height coordinate (Figure 5(c)) was within a range of ±5.0 cm.

Table 3 shows statistical indicators of the positioning errors of all test points (including the sample point as shown in Figure 4(a–c)). The levels of north and east errors were higher than the accuracy levels of the normal RTK-GPS positioning method. The measurements of 2 cm or lesser errors accounted for approximately 99% over all test points. As for the ellipsoid height, the measurements of 4 cm or lesser errors accounted for approximately 89% over all test points, considered satisfactory. However, the maximum error was somewhat large, and this will be addressed in another study of cause analysis and accuracy improvement.

**Figure 6.**
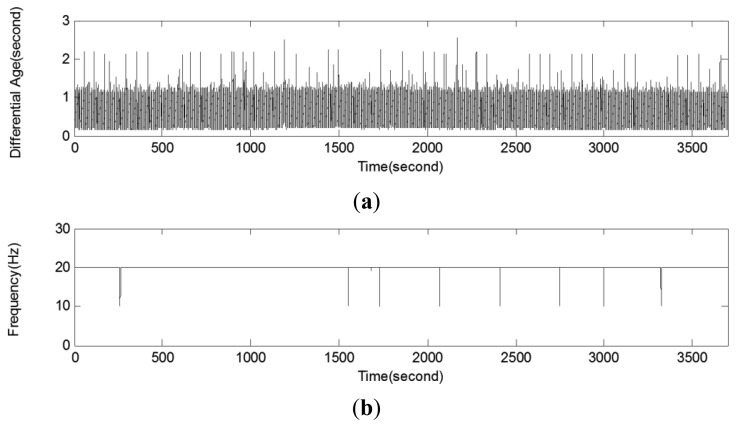
A Sample of test results (at Point 1) of performance evaluation. (**a**) Latency until the correction message transferred from the fixed station. (**b**) Positioning frequency.

Figure 6 shows the latency required to transfer the correction message and positioning frequency of the NRTK application using the one-hour data at one fixed point as a sample. Figure 6(a) shows the latency of the correction message, with the NRTK positioning point as the reference. Most of them are less than two seconds for all test points, within a range of 0.78 ± 0.36 s. Because precision positioning is possible when the latency of the GPS correction message is within a few seconds [13], this value is quite satisfactory. The smartphone for the study supported 3G mobile communication. The test results showed that a 3G or 4G smartphone is suitable for the reception of the NRTK positioning correction message. Figure 6(b) shows the positioning frequency at the sample point. The measured coordinates were precise at 20 Hz across sections, except for approximately eight times in an hour. This showed that the NRTK application for smartphones is suitable for precision positioning with high frequency.

## 4. Determination of the Error Model for the NRTK Positioning Results

The NRTK positioning method is used in a wide range of areas, including geodetic survey [5], structure displacement measurement [3], and unmanned vehicles [14]. These application areas estimate more accurate positions and posture values, by applying a state estimation filter such as the Kalman Filter [2,3,15–17] to the NRTK positioning coordinate, instead of using the direct NRTK measurements. This study proposed a method for determining the error model of the NRTK positioning coordinate, applied to the state estimation filter and the developed error model and Kalman Filter were included in the NRTK application. The reason for this is that the accurate determination of the error model for the NRTK positioning results is important, to ensure the performance of the state estimation filter and to get more precise positioning results.

The 3D absolute coordinate measured using the RTK-GPS positioning method is very accurate, with an error as large as several centimeters. The error statistically represents the characteristic of the random error. However, Schwieger [18] suggests that the values measured at a certain time have a high correlation in the case of the RTK-GPS measurements at 1 Hz or higher, and that they lead to autocorrelation and random errors. Based on previous study results, this study now presents a determination method for an error model of NRTK measurement, considering the systematic error due to random error and autocorrelation.

**Figure 7.**
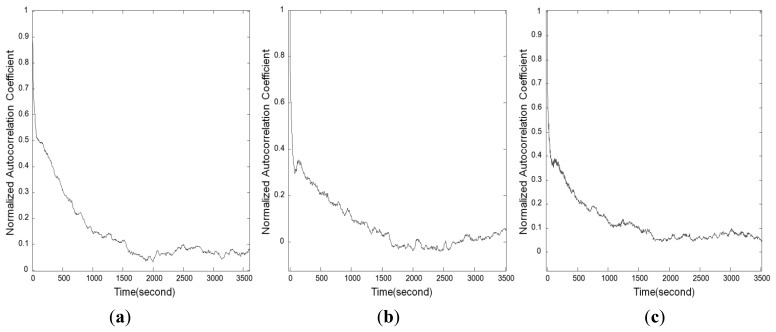
Correlograms for the elements of the 3D coordinates measured via the NRTK positioning method. (**a**) North coordinates. (**b**) East coordinates. (**c**) Ellipsoidal heights.

The correlogram analysis, which has the autocorrelation coefficient marked on the time domain, helps determine whether the NRTK measurements include the autocorrelation error. If the *N* measurements in the time series are *l*_1_,*l*_2_,*l*_3_, …, *l_k_*, …, *l_N_*, the time difference between measurements is Δ*t*. The average of measurements is *M*, while the autocovariance coefficient of the measurements *Ĉ*(*i*) is expressed in Equation (1), and autocorrelation coefficient in Equation (2) [19]:

(1) $$\begin{array}{cc} \hat{C}(0)= & \sum_{k=1}^{N}\left(l_{k}-M)(l_{k}-M)/(N-1\right)\\ \hat{C}(1)= & \begin{array}{c} \sum_{k=2}^{N}\left(l_{k}-M)(l_{k-1}-M)/(N-2\right)\\ \vdots \end{array}\\ \hat{C}(i)= & \sum_{k=i+1}^{N}\left(l_{k}-M)(l_{k-i}-M)/(N-i-1\right) \end{array}$$

(2) $$R_{i}=\frac{\hat{C}(i)}{\hat{C}(0)}$$

where *i* is the order of the autocorrelation coefficient, and the time difference between *l_k_* and *l_k_*_−1_ is *i* × Δ*t*. The autocorrelation coefficient for each *i* can be calculated from the RTK-GPS values measured for a long period at a fixed point. A diagram showing the correlation between *i* and *R_i_* is called a correlogram. Figure 7 shows the correlogram drawn by the analysis of the coordinates measured at 2 Hz for more than an hour, using the NRTK application. The figures indicate a distinct tendency of the autocorrelation error. The correlations among the values within a time difference of 10 s were 0.5 or greater, in each of the north, east, and ellipsoid height coordinates. The correlations decreased as the time difference among measurements increased, while the autocorrelation error had an effect within a range of approximately five minutes.

In Figure 7, it is seen that the autocorrelation coefficient distribution of the NRTK observation values follows the Gauss-Markov process. Accordingly, the autocorrelation function can be expressed as an exponential function as follows:

(3) $$R_{l}(i)=e^{-\alpha \cdot i\cdot \Delta t}$$

where *α* is a coefficient representing the degree of correlation. If the standard deviation of the random error in NRTK measurements is *σ_δ_*, and the standard deviation of the autocorrelation error is *σ*_Δ_, then the statistical error model of the NRTK measurements is expressed in Equation (4) [20].

(4) $${\left(\sum_{ll}\right)}_{n\times n}=\left[\begin{array}{cccc} \sigma_{\delta }^{2}+\sigma_{\Delta }^{2}\cdot e^{-\alpha \cdot 0\cdot \Delta t} & \sigma_{\Delta }^{2}\cdot e^{-\alpha \cdot 1\cdot \Delta t} & \cdots & \sigma_{\Delta }^{2}\cdot e^{-\alpha \cdot (n-1)\cdot \Delta t}\\ \sigma_{\Delta }^{2}\cdot e^{-\alpha \cdot 1\cdot \Delta t} & \sigma_{\delta }^{2}+\sigma_{\Delta }^{2}\cdot e^{-\alpha \cdot 0\cdot \Delta t} & \cdots & \vdots \\ \vdots & \vdots & \ddots & \sigma_{\Delta }^{2}\cdot e^{-\alpha \cdot 1\cdot \Delta t}\\ \sigma_{\Delta }^{2}\cdot e^{-\alpha \cdot (n-1)\cdot \Delta t} & \cdots & \sigma_{\Delta }^{2}\cdot e^{-\alpha \cdot 1\cdot \Delta t} & \sigma_{\delta }^{2}+\sigma_{\Delta }^{2}\cdot e^{-\alpha \cdot 0\cdot \Delta t} \end{array}\right]$$

The parameters that must be determined, to apply the error model in Equation (4) to the filtering of NRTK measurements, are *σ_δ_*, *σ*_Δ_ and *α*. These values can be estimated using the variance of the sample mean via the least square method. Meier and Keller [21] summarized the correlation between the variance of the sample mean and *σ_δ_*, *σ*_Δ_ and *α*, as in Equation (5).

If the variances of the sample mean by sample size (*n*) are calculated using the data measured at the same point for a long period, three unknown elements that represent the error model can be estimated via the least square method:

(5) $$\sigma_{M}^{2}=\frac{1}{n}\sigma_{\delta }^{2}+\frac{1}{n}\sigma_{\Delta }^{2}+\frac{2}{n^{2}}\sum_{k=1}^{n-1}(n-k)\cdot \sigma_{\Delta }^{2}\cdot e^{-\alpha \cdot k\cdot \Delta t}$$

**Figure 8.**
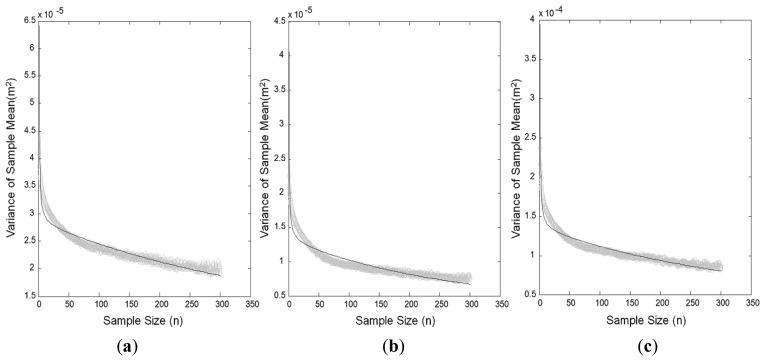
Variances of the sample mean were calculated from the observation values and estimated using the least square method. (**a**) North coordinates. (**b**) East coordinates. (**c**) Ellipsoidal heights.

Table 4 shows the unknown elements of the error model, estimated using the NRTK coordinates measured from a fixed point for six hours by component. Figure 8(a–c) shows Equation (5) by the component in an overlapping manner, determined using the variance of the sample mean calculated from the measured coordinates, and the values estimated via the least square method.

The static measurement results were filtered, to evaluate the effectiveness of the error model for the NRTK application that is calculated considering the random and autocorrelation errors. The test results were classified into two cases: *Case 1* with an error model that only considered random errors, and *Case 2* with an error model that considered both random and autocorrelation errors. The 3D coordinates that were measured using the NRTK application were filtered, while their accuracies were comparatively analyzed.

**Figure 9.**
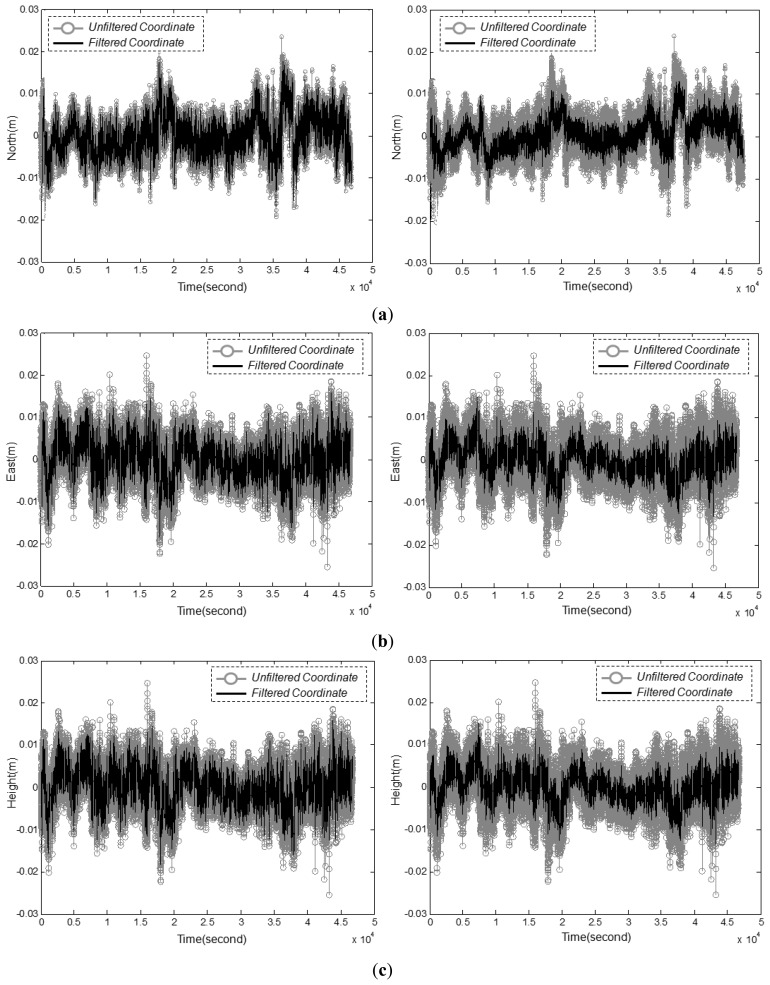
Filtering results of NRTK positioning for the 3D coordinates. (**a**) North coordinates from *Case 1* (left) and *Case 2* (right). (**b**) East coordinates from *Case 1* (left) and *Case 2* (right). (**c**) Ellipsoidal heights from *Case 1* (left) and *Case 2* (right).

Figure 9(a–c) shows the time series data for the north, east, and ellipsoid height coordinates, respectively. The static coordinates measured for a long period, and filtering results with the Kalman Filter, were superposed. The left and right figures in Figure 9 represent the results of *Case 1* and *Case 2*, respectively. Also, Table 5 shows the statistical indicators of filtering results of *Case 1* and *Case 2*. It can be seen in Figure 9 and Table 5, the test results show that with the method proposed in this study, the accuracy is higher in *Case 2* than in *Case 1*.

## 5. Conclusions

In this study, an NRTK application for smartphones was developed, and its performance evaluated via tests. A method for determining the error model of the measured coordinates was proposed, to use the NRTK measurements with higher accuracy, using dynamic state estimation filters. The study results are summarized as follows:

- The advantages of smartphones were used to develop a NRTK application with wireless communication, NTRIP client, and RTK processing functions. The composition of the NRTK measurement system was thus simplified, while the NRTK measurement system was put together without using an expensive GPS receiver with RTK processing functions.
- The evaluation of the positioning accuracy of the NRTK application showed that the RMSEs of the north, east, and ellipsoid height coordinates were very precise (0.0069 m, 0.0068 m, and 0.0269 m, respectively), when the NRTK positioning was conducted at a high frequency (20 Hz).
- The latency for correction message transfer, which significantly affected the accuracy of the NRTK measurements, was 0.71 ± 0.32 s. This confirmed that 3G smartphones are very suitable for NRTK positioning.
- A method for determining the error model of NRTK application positioning results was presented. This method aimed to use the NRTK application positioning results more accurately, via state estimation filters. An error model was developed (by considering both the random error and autocorrelation error), and was applied to the Kalman Filter. The positioning results were more accurate, compared with those from an error model that only considered random errors.

## Figures and Tables

**Table 1.** Main functions of the application and considerations by functions.

**Main Functions**	**Detailed Function**	**Remark**
NTRIP client (SOCKET communication)	- NTRIP broadcaster connection and user authentication - NRTK corrective message reception - User position data transmission	The time required to transfer the correction message must be short
NRTK positioning	- Enter the GPS observation value - Enter the NRTK correction message - Check NRTK positioning	Real-time positioning must be possible at 20 Hz or higher GPS observation values and correction messages in diverse formats must be recognized
GPS receiver control and Data reception (Bluetooth communication)	- Receive GPS observation value - GPS receiver control (positioning frequency and interface format)	A control function suitable for the communication protocol of the GPS receiver is required
Utility	- Save and retrieve position data - Set out and other functions	Scalability for meeting the diverse requirements of the user is required

**Table 2.** Application development environment.

**Item**	**Detail**
OS	Android 2.2 (Froyo)
SDK	Android API Level 8 & JDK 1.7
Integrated Development Environment	Eclipse
Programming Language	Java

**Table 3.** Statistical indicators of the positioning accuracy of the NRTK application calculated from the test results at twelve fixed points displayed in Figure 4.

**Statistical Indicator**	**North Coordinates**	**East Coordinates**	**Ellipsoid Heights**
RMSE	0.69 cm	0.68 cm	2.69 cm
Average deviation	0.56 cm	0.55 cm	2.12 cm
Maximum deviation	2.86 cm	2.77 cm	15.12 cm
Ratio of accurate measurements	99.12% (within 0.02 m deviation)	98.91% (within 0.02 m deviation)	89.66% (within 0.04 m deviation)

**Table 4.** Error model applied to the NRTK application positioning results.

**Error Model**		***σ_δ_***	***σ*_Δ_**	***α***
Coordinate Component	North	0.60 cm (±0.0122 cm)	0.53 cm (±0.0014 cm)	0.004587 s^−1^ (±0.000176 s^−1^)
	East	0.53 cm (±0.0117 cm)	0.36 cm (±0.0016 cm)	0.008014 s^−1^ (±0.000395 s^−1^)
	Height	1.62 cm (±0.0281 cm)	1.15 cm (±0.0037 cm)	0.005921 s^−1^ (±0.000259 s^−1^)

**Table 5.** Statistical indicators of the filtering effects for use of random and autocorrelation error model in NRTK application.

**Statistical Indicator**	**North Coordinates**		**East Coordinates**		**Ellipsoid Heights**	

	***Case 1***	***Case 2***	***Case 1***	***Case 2***	***Case 1***	***Case 2***
RMSE	0.65 cm	0.47 cm	0.44 cm	0.32 cm	1.41 cm	1.06 cm
Average deviation	0.58 cm	0.31 cm	0.55 cm	0.24 cm	1.87 cm	1.02 cm
Maximum deviation	2.91 cm	2.03 cm	1.89 cm	1.34 cm	6.73 cm	4.74 cm
Ratio of accurate measurements	99.21%	99.97%	99.35%	100.00%	91.74%	97.66%
	*(within 0.02 m deviation)*		*(within 0.02 m deviation)*		*(within 0.04 m deviation)*	

## References

[1] Weber G., Dettmering D., Gebhard H. (2005). Networked transport of RTCM via Internet Protocol (NTRIP). Int. Assoc. Geodesy Symp..

[2] Lee H.K. (2010). An integration of GPS with INS sensors for precise long-baseline kinematic positioning. Sensors.

[3] Hwang J., Yun H., Park S.-K., Lee D., Hong S. (2012). Optimal methods of RTK-GPS/Accelerometer integration to monitor the displacement of structures. Sensors.

[4] Al-Shaery A., Lim S., Rizos C. (2011). Investigation of different interpolation models used in Network-RTK for the virtual reference station technique. J. GPS.

[5] Rizos C. (2002). Network RTK research and implementation: A geodetic perspective. J. GPS.

[6] Trajkovski K.K., Sterle O., Stopar B. (2010). Study positioning with high sensitivity GPS sensors under adverse conditions. Sensors.

[7] Bruyninx C., Habrich H., Söhne W., Kenyeres A., Gstangl G., Völksen C. (2012). Enhancement of the EUREF permanent network services and products. Int. Assoc. Geodesy Symp..

[8] Martín A., Padín J., Anquela A.B., Sánchez J., Belda S. (2009). Compact integration of a GSM-19 magnetic sensor with high-precision positioning using VRS GNSS technology. Sensors.

[9] Park Y., Chen J.V. (2007). Acceptance and adoption of the innovative use of smartphone. Industrial Manage. Data Syst..

[10] Retscher G. (2002). Accuracy performance of virtual reference station (VRS) networks. J. GPS.

[11] Tamura Y., Matsui M., Pagnini L.C., Ishibashi R., Yoshida A. (2002). Measurement of wind-induced response of buildings using RTK-GPS. J. Wind Eng. Ind. Aerodyn..

[12] NovAtel Inc. (2006). OEMV Family Installation and Operation Manual.

[13] Weber G. Streaming Real Time IGS Data and Products Using NTRIP.

[14] Uradziński M., Langley R.B., Kim D. The Usefulness of Internet-Based (Ntrip) RTK for Navigation and Intelligent Transportation Systems.

[15] Kalman R.E. (1960). A new approach to linear filtering and prediction problems. Trans. ASME.

[16] Welch G., Bishop G. An Introduction to the Kalman Filter. http://www.cs.un-c.edu/welch/kalman/kalmanIntro.html.

[17] Gomez-Gil J., Alonso-Garcia S., Gómez-Gil F.J., Stombaugh T. (2011). A simple method to improve autonomous GPS positioning for tractors. Sensors.

[18] Schwieger V. The Effect of Interepochal Correlations in the Analysis of Monitoring Surveys.

[19] Strang G., Borre K. (1997). Linear Algebra, Geodesy, and GPS.

[20] Kuhlmann H. Kalman-Filtering with Coloured Measurement Noise for Deformation Analysis.

[21] Meier S., Keller W. (1990). Geostatistik (Geostatistic).

